# Why is the recognition rate of psychological distress under-estimated in general hospitals? A cross-sectional observational study in China

**DOI:** 10.1097/MD.0000000000016274

**Published:** 2019-07-05

**Authors:** Yu Wang, Alexandra M. Murray, Anne-Kristin Toussaint, Liang Chen, Wan-Jun Guo, Ning He, Shan-Xia Luo, Jian-Ying Yu, Yang Liu, Ming-Jin Huang, Zai-Quan Dong, Lan Zhang

**Affiliations:** a Mental Health Center, West China Hospital of Sichuan University, Chengdu, Sichuan, People's Republic of China; b Department of Psychosomatic Medicine and Psychotherapy, University Medical Center Hamburg-Eppendorf, Germany.

**Keywords:** aassociated factors, concordance, doctors, inpatients, psychological distress, recognition

## Abstract

This study aimed to investigate the recognition rate of psychological distress in general hospitals in China and to examine the main associated factors.

Using a cross-sectional study design, the questionnaires were administered to a total of 1329 inpatients from a tertiary hospital. The Patient Health Questionnaire-9 (PHQ-9), the Generalized Anxiety Disorder 7-item scale (GAD-7), the Patient Health Questionnaire (PHQ-15) and the Whiteley-7 (WI-7) were used to assess patients’ mental health status. Two subjective questions were used to identify the awareness of psychological distress in patients and doctors.

The frequency of psychological distress measured by the questionnaires was high in our sample (53.4%). However, the recognition rates of both patients (34.9%) and by doctors (39.1%) was low. The concordance rate between patients and doctors of whether the patient had psychological distress or not was extremely poor (Kappa = 0.089, *P* = .001). Factors associated with the poor concordance rate included patients’ annual household income and clinically significant self-reported symptoms of anxiety and hypochondriasis.

The recognition rate of psychological distress was underestimated and this may be related to a lack of awareness of mental disturbances and patients’ low annual household income.

## 1 Introduction

Mental distress is common.^[1–3]^ It seriously affects patients’ quality of life as well as the course and prognosis of physical diseases.^[4]^ Depression and anxiety are the 2 most common mental disorders in the general population. In 2001 to 2005, a large epidemiological study demonstrated that mood and anxiety disorders were the most frequent mental disorders in China, with a prevalence of 6.1% and 5.6%, respectively.^[5]^

Patients with multiple somatic symptoms comprise a substantial proportion of patients in different health care settings.^[6–7]^ Studies have shown that up to 9% of patients in clinics for general medical practice and up to 5% of the general population meet the diagnostic criteria for hypochondriasis and that approximately 10% of primary care patients meet the diagnostic criteria for somatic symptom disorder (SSD).^[3,8–9]^ Because SSD is one of the most difficult diseases to diagnose, some doctors are likely to provide unnecessary diagnostic procedures and treatment to avoid overlooking medical disease.^[10–11]^ However, excessive interventions may foster somatic fixations of patients, leading to severe functional impairment and increased health care costs.^[3,12–14]^ Given the substantial economic loss due to psychological distress, it is important that they are recognized early so that appropriate treatment can be initiated.^[15–17]^

The recognition rate of mental disorders in general hospitals is very low. In 2008, Liu found a recognition rate of only 3.33% for general practitioners in China, which is significantly Löwer than in Western countries.^[18–19]^ The survey also showed that only 5.2% of patients with anxiety were identified by general practitioners. With the development of psychosomatic medicine in China, later studies conducted in Beijing (21.0%) and Shanghai (18.5%) reported that the identification rate of depression by clinical physicians was higher than previously reported.^[20–21]^

Critically, in 2009, Phillips reported that only 5% of all people with a diagnosable mental illness had ever seen a mental health professional.^[5]^ If we consider a diagnosis as a pathway to receiving appropriate treatment, then it is very important to understand the factors that influence physicians’ recognition of mental disorders. On the other hand, patients who recognize their own mental problems may be more likely to be motivated to undergo treatment.^[22]^ Ultimately, it may be the concordance between physician and patient recognition that is the most beneficial for patient care. Previous studies have suggested that both psychiatric outpatients and mental health professionals view the concept of concordance positively and that in GP settings, medication compliance is higher, with higher levels of patient-physician concordance.^[23–24]^ However, there are many differences between Chinese and Western perceptions of mental health, and expectations of care.^[25]^ Few studies in China have focused on the concordance of recognition of psychological distress between doctors and patients themselves, as well as factors related to concordance in China.

To investigate this issue, we conducted a cross-sectional survey in the West China Hospital of Sichuan University. Our research aim was to investigate the following research questions:

(1) the frequency of mental distress among Chinese patients hospitalized in general hospitals;
(2) the recognition rate of mental distress in patients and doctors;
(3) the concordance rate between patients and doctors; and
(4) the factors associated with the concordance.

## 2 Materials and methods

### 2.1 Sample

We conducted the survey on a random day in October 2013. Participants in this study were inpatients recruited from 10 departments (Oncology, Cardiology, Respiratory Medicine, Rehabilitation, Geriatrics and Gerontology, General Practice, Pain Management, Thyroid and Breast Surgery, Rheumatology, and Hepatic Surgery) in the West China Hospital of Sichuan University. These departments cover medical and surgical diseases, chronic and acute diseases, neoplastic and non-tumour diseases, short-term and long-term hospitalization, and low and high expenditures. They greatly represent the different psychological conditions of hospitalized patients in our hospital.

All inpatients of these departments were potential participants in our study. The following inclusion criteria were used:

1. inpatient from the selected wards;
2. sufficient language skills to understand the questionnaires; and
3. provided informed consent to participate in the study.

The exclusion criteria were:

1. discharged from the hospital on the day of survey;
2. inability to finish the self-reported questionnaire on their own because of serious physical condition or mental status.

All doctors in charge of these participants were included in our study. All data were collected by investigators who were well-trained medical doctors, nurses, or medical students. The study was approved by the Ethics Committee of West China Hospital of Sichuan University. Written informed consent was obtained from all participants.

### 2.2 Instruments

#### 2.2.1 Demographic questionnaire

We designed a sociodemographic questionnaire to collect information including patients’ gender, marital status, ethnicity, educational background, and annual household income. We also ascertained the doctors’ gender, years of work experience and educational background. In addition to the demographic items, patients completed the following questionnaires.

#### 2.2.2 Scales

**The Patient Health Questionnaire-9.**

The Patient Health Questionnaire-9 (PHQ-9) is a 9-item questionnaire focusing on depression. The PHQ-9 total score ranges from 0 to 27. In the present study, we used a score ≥ 10 as the cut-off point for the clinical significance of depression.^[26–29]^ The Generalized Anxiety Disorder 7-item scale (GAD-7) was used to evaluate symptoms of anxiety and a score ≥ 10 represented clinically significant anxiety in the present study.^[30–32]^ Somatic symptoms were assessed by the Chinese version of the PHQ-15, and a score of ≥ 10 was used because it has been shown to be the optimal cut-off value to predict the diagnosis of a somatoform disorder in primary care patients.^[33–34]^ The Whiteley-7 (WI-7) scale was developed to screen for hypochondriasis.^[35–36]^ The WI-7 allows for a dichotomous choice of “yes” or “no”. A score of “1” was given for each “yes” response, resulting in a sum score ranging between 0 and 7.^[37–38]^ The cut-off value yielding the maximum Youden index for the WI-7 was ≥ 3.^[39–40]^ The result of those scales was applied as the gold standard for judging both the recognition of doctors and the self-awareness of the psychological problems of patients. Once the patients scored above the cut-off in any scale of the questionnaire set, we took the result to be positive.

#### 2.2.3 In addition to the standardized questionnaires, we developed questions to measure the awareness of psychological distress by both, patients and doctors

Question 1 (to patients): Do you think that you are suffering from any psychological problems or emotional problems?

Question 2 (to doctors): Do you think that the patient is suffering from any psychological problems or emotional problems?

When the answer to Question 1 or Question 2 was “Yes”, we believed that patients or doctors (respectively) thought that the patient was suffering from psychological distress. Doctor-patient consistency was defined as the doctor and patient having the same answer to the 2 questions.

### 2.3 Data analysis

Patients were regarded to be suffering from psychological distress when they scored ≥ 10 on PHQ-9, PHQ-15, or GAD-7 or ≥3 on Whiteley-7. For simplicity, these variables were dichotomized for future analyses. Regardless of the questionnaire results, responses were regarded as concordant if both patients and doctors agreed that the patients suffered from psychological problems (or not). SPSS 18.0 was used for data analysis, and statistical significance was set at *P* < .05 based on two-tailed tests.

Descriptive statistics were used to describe the frequency of depression, anxiety, somatization, and hypochondriasis as measured by the questionnaires and to analyse the recognition rate of psychological distress by patients and doctors as well as the concordance rate of identification. Concordance between doctors and patients was assessed via kappa statistics. Chi-Squared analyses were conducted to investigate the different concordance rates between the socio-demographic variables and clinically significant depression, anxiety, somatization, or hypochondriasis symptom severity. Binary logistic regression was performed to explore the potential influencing factors of the concordance rates on the recognition of psychological distress between doctors and patients. The concordance rate was taken as the dependent variable and variables that were significant in the univariate analyses were taken as the independent variables in the binary logistic regression.

Because some doctors filled out multiple questionnaires, which may have affected the independence of the observations, we randomly selected 1 questionnaire per doctor to form a new sample to assess the concordance between doctors and patients.

## 3 Results

Of 1662 inpatients approached in 10 departments, 151 patients were excluded based on the exclusion criteria and 149 patients refused to participate in the study. The main reasons that patients gave in cases of non-participation were lack of time (n = 27) or interest (n = 60). The final sample consisted of 1362 patients, with an overall response rate of 90.1%. All doctors in charge of these patients filled out the questionnaires, and 361 doctors were involved in the survey (response rate of 100.0%). Cases with more than 15% missing data were excluded. Therefore, 1329 eligible patients were included in our study. According to the inclusion and exclusion criteria, nearly 3300 patients throughout the hospital should have been included in the study. In fact, 40.3% of eligible patients (1329/3300) were included in our study, so we believe that the sample strongly represents the hospital's annual patient population. The subsample used to assess the concordance included 361 questionnaires. Other socio-demographic data are shown in Table 1.

**Table 1:**
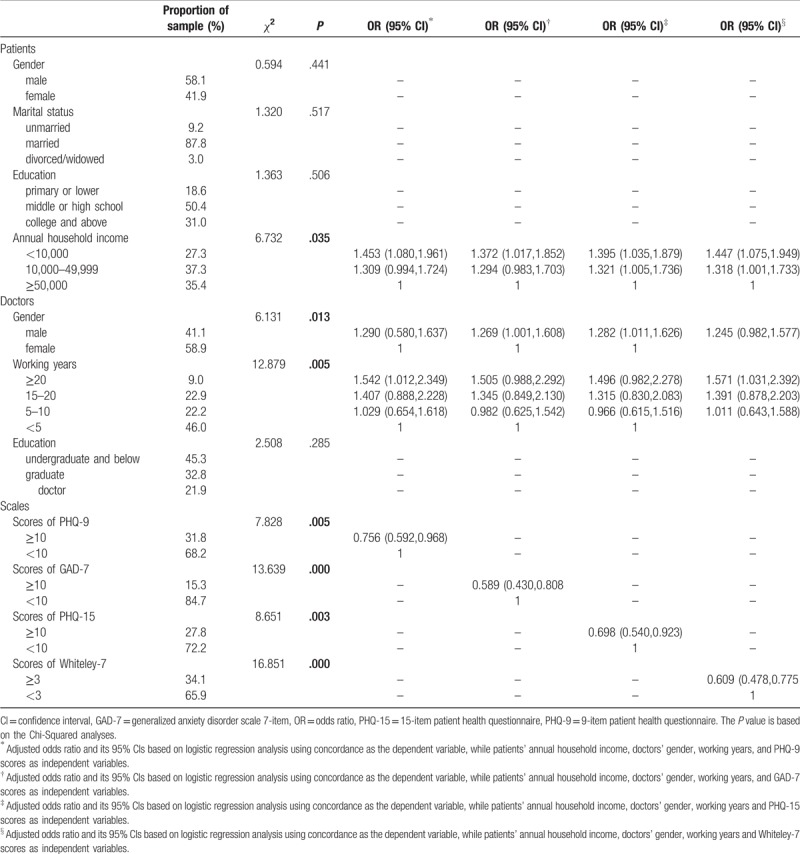
Demographic data and associations of the concordance between patients and doctors with sociodemographic variables, hypochondriasis, somatization, depression, and anxiety in the sample 1 (N = 1329).

Among the total N = 1329 patients, 53.4% (n = 710) of participants reported psychological distress as measured by the standardized questionnaires (PHQ-9, GAD-7, PHQ-15 and Whiteley-7), of which 31.8% (n = 423) exceeded the cut-off score for depression, 15.3% (n = 204) for anxiety, 27.8% (n = 369) for somatization and 34.1% (n = 453) for hypochondriasis. In total, 34.9% (n = 462) of patients thought that they were suffering from psychological or emotional problems; however, doctors reported this to be the case in 39.1% (n = 505) of patients. Patients accurately recognized their own psychological or emotional problems in 65.20% (n = 862) of cases, and doctors did so in 55.12% (n = 711). The sensitivity was 50.14% (n = 348) in the patient group and 44.56% (n = 303) in the group of practitioners; the specificity was 81.85% (n = 514) in the patient group and 66.89% (n = 408) in the group of practitioners. The concordance rate of the recognition of patients’ psychological or emotional problems between patients and doctors was low (Kappa = 0.089, *P* = .001) and was lower (Kappa = 0.067, *P* = .198) in the smaller subsample (N = 361). The details for this sample are shown in Table 2.

**Table 2:**
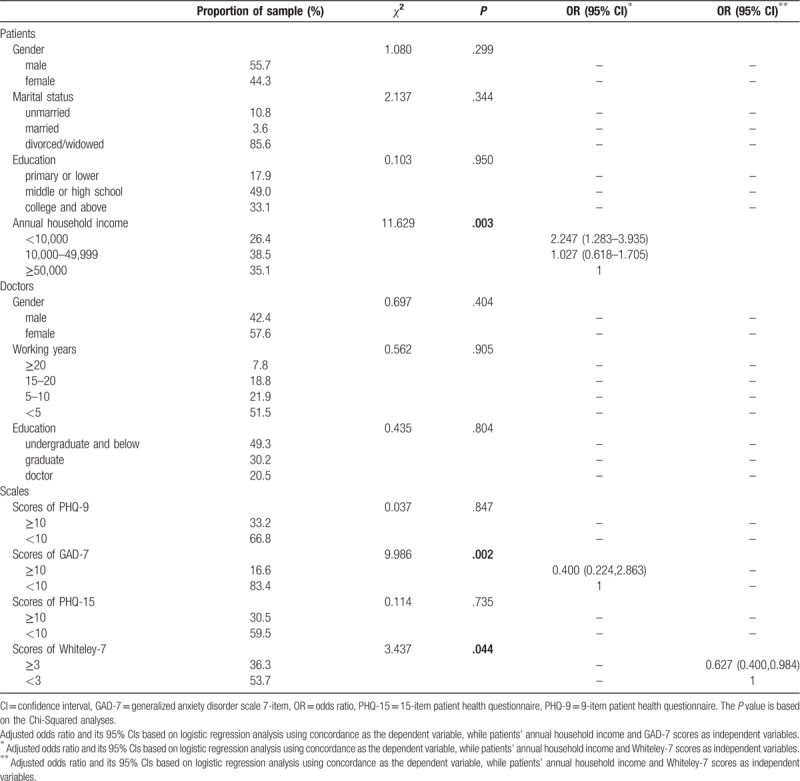
Demographic data and associations of the concordance between patients and doctors with sociodemographic variables, hypochondriasis, somatization, depression, and anxiety in the new sample (N = 361).

The concordance of patients’ and doctors’ identification of psychological problems differed according to doctors’ gender and working years as well as patients’ annual household income and symptoms of clinically significant depression, anxiety, somatization, and hypochondriasis as measured by the self-report scales (Table 1). In the next step, we included the variables that were significant (*P* < .05) in the univariate analyses in binary logistic regression analyses. The factors associated with the poor concordance rate included patients’ annual household income and clinically significant self-reported symptoms of anxiety and hypochondriasis in the subsample (N = 361). Patients with a low annual household income were less likely to have a congruent identification with doctors. We also found that patients with anxiety or hypochondriasis tended to have higher concordance with doctors (Table 2).

## 4 Discussion

This is one of the first studies to combine validated psychometric tools and self-reported patient and doctor insights to investigate psychological or emotional problems in non-psychiatric hospitalized patients in China. We found that the frequency of psychological or emotional problems measured by self-rated scales was as high in our study as in previous studies, so it is reasonable to believe that this sample is representative, and that the results are generalizable.^[5,8–9,18,38]^ Nevertheless, a large percentage of patients and doctors showed a lack of awareness of mental disturbances and did not accurately identify psychological or emotional problems.^[5,18]^ More importantly, the concordance rate of the recognition of mental distress between patients and physicians was extremely poor. This could be due to their poor knowledge about psychological distress and barriers in communication of these factors. Such findings corroborate with studies of Chinese-speaking Australians, suggesting that mental health literacy is poor in this community and that Chinese people would prefer not to seek professional help for psychological problems due to stigma or other reasons.^[41–42]^ The relatively high rate of somatization in this sample may also explain the relatively poor rates of concordance. Previous studies suggest that those who expressed their mental distress in “psychological” ways relied on themselves or family for help, whereas those who “somatised” sought medical help.^[43]^ If Chinese patients did not expect their physician to assist with psychological matters, they were unlikely to communicate their mental distress to their physicians.

Both models suggest that patients who had a low annual household income were less likely to have a congruent identification with doctors. These patients were likely to have a poor educational background, low social status, and little money, so they were more likely to ignore mental health problems and complain less about psychological distress to the doctor than patients with a high income. On the other hand, patients who were suffering from anxiety and hypochondriasis had more psychological complaints, so they were more easily detected by the doctors. However, in model 1 (N = 1329), we also found that doctors with less working experience and female doctors were more likely to have a congruent identification with patients compared to doctors with more working experience and male doctors. They might pay more attention to mental health and have more patient and contact time to provide patients with more mental health-related support, so they might be more likely to observe and report psychological or emotional problems. This result is similar to the results of previous studies, which reported that patients who have a good relationship with their doctor tended to report their psychological distress.^[44]^ The different results between the 2 models may be associated with the gathering effect of a doctor who observed multiple patients in model 1 (N = 1329), while doctors and patients were paired one-to-one in a special subsample (N = 361).

Of course, some other factors that we did not include in this study may be relevant to patient-doctor concordance. Since psycho-somatic medicine is only starting to develop in China, medical workers in non-psychiatric settings have little or no training in mental health, so they may be unable (and often unwilling) to recognize the mental problems in their patients and provide basic psychiatric services. There is also a lack of reliable, effective and efficient psychological measurement tools to help doctors and nurses screen patients for psychological distress. There is a lack of scientific and large-sample investigations focusing on inpatients’ mental health conditions. Another important problem is that patients in China tend to ignore their mental problems because of a strong fear of being stigmatized.

Our research has several limitations. First, there is no evidence of the psychometric quality of the utilized questions on the awareness of psychological problems. Therefore, we could only obtain rough results and trends. Second, our study lacked the gold standard for the diagnosis of psychological disorders because no diagnostic interviews were conducted. In addition, unmeasured and unknown confounders might have influenced the results in our study.

## 5 Conclusion

The frequency of elevated symptoms of depression, anxiety, somatization, and hypochondriasis was high in our sample of hospitalized patients, but a large percentage of patients and general doctors did not accurately identify psychological problems in patients. To improve the diagnostic process and treatment for mental disorders, we should encourage doctors to spend more time and energy investigating patients’ concerns, conduct more training on mental health in general hospitals and offer more reliable, effective and efficient psychological measurement tools to help general doctors screen their patients. At the same time, we should provide patients with more psychological health education to help include mental health literacy to assist in the recognition of mental disturbances and encourage them to seek professional psychological support without being discouraged by stigma. Finally, more longitudinal research using the diagnostic gold standard on this topic are necessary in the future.

## Acknowledgments

The authors thank Prof. Kang (Evidence-based Medicine Center of Sichuan University) for his guidance in statistical analysis.

## Author contributions

**Conceptualization:** Yu Wang, Liang Chen, Wan-Jun Guo, Ning He, Shan-Xia Luo, Jian-Ying Yu, Yang Liu, Ming-Jin Huang, Zai-Quan Dong, Lan Zhang.

**Data curation:** Yu Wang, Liang Chen, Yang Liu, Ming-Jin Huang.

**Formal analysis:** Yu Wang, Alexandra M. Murray, Anne-Kristin Toussaint.

**Investigation:** Yu Wang, Liang Chen, Shan-Xia Luo, Jian-Ying Yu, Yang Liu.

**Methodology:** Yu Wang, Alexandra M. Murray, Anne-Kristin Toussaint, Liang Chen, Wan-Jun Guo.

**Project administration:** Yu Wang.

**Resources:** Yu Wang, Ning He, Shan-Xia Luo, Jian-Ying Yu, Yang Liu, Ming-Jin Huang, Zai-Quan Dong.

**Software:** Yu Wang, Alexandra M. Murray, Anne-Kristin Toussaint.

**Supervision:** Lan Zhang.

**Validation:** Yu Wang, Alexandra M. Murray, Anne-Kristin Toussaint, Ning He.

**Visualization:** Yu Wang, Alexandra M. Murray, Anne-Kristin Toussaint.

**Writing – original draft:** Yu Wang.

**Writing – review & editing:** Alexandra M. Murray, Lan Zhang.

## References

[1] Zhong BL Chen HH Zhang JF Prevalence, correlates and recognition of depression among inpatients of general hospitals in Wuhan, China. Gen Hosp Psychiat 2010;32:268–75. 10.1016/j.genhosppsych.2010.01.016 20430230

[2] Ying DG Jiang S Yang H Frequency of generalized anxiety disorder in Chinese primary care. Postgrad Med 2010;122:32–8. 20675969 10.3810/pgm.2010.07.2173

[3] Löwe B Spitzer RL Williams JB Depression, anxiety and somatization in primary care: syndrome overlap and functional impairment. Gen Hosp Psychiat 2008;30:191–9. 10.1016/j.genhosppsych.2008.01.001 18433651

[4] Cole M McCusker J Ciampi A The prognosis of major and minor depression in older medical inpatients. Am J Geriat Psychiat 2006;14:966–795. 10.1097/01.JGP.0000224327.16963.9f 17068319

[5] Phillips MR Zhang J Shi Q Prevalence, treatment, and associated disability of mental disorders in four provinces in China during 2001-05: an epidemiological survey. Lancet 2009;373:2041–53. 19524780 10.1016/S0140-6736(09)60660-7

[6] Toft T FINK P Oernboel E Mental disorders in primary care: prevalence and co-morbidity among disorders. Results from the Functional Illness in Primary care (FIP) study. Psychol Med 2005;35:1175–84. 16116943 10.1017/s0033291705004459

[7] Creed FH Davies I Jackson J The epidemiology of multiple somatic symptoms. J Psychosom Res 2012;72:311–7. 22405227 10.1016/j.jpsychores.2012.01.009

[8] Creed F Barsky A A systematic review of the epidemiology of somatisation disorder and hypochondriasis. J Psychosom Res 2004;56:391–408. 15094023 10.1016/S0022-3999(03)00622-6

[9] Gureje O Ustün TB Simon GE The syndrome of hypochondriasis: a cross-national study in primary care. Psychosom Med 1997;27:1001–10. 10.1017/s0033291797005345 9300506

[10] Dimsdale JE Dantzer R A biological substrate for somatoform disorders: importance of pathophysiology. Psychosom Med 2007;69:850–4. 18040093 10.1097/PSY.0b013e31815b00e7 PMC2908292

[11] Bensing JM Verhaak PF Somatisation: a joint responsibility of doctor and patient. Lancet 2006;367:452–4. 16473108 10.1016/S0140-6736(06)68155-5

[12] Biderman A Yeheskel A Herman J Somatic fixation: the harm of healing. Soc Sci Med 2003;56:1135–8. 12593884 10.1016/s0277-9536(02)00108-9

[13] Tomenson B Essau C Jacobi F Total somatic symptom score as a predictor of health outcome in somatic symptom disorders. Br J Psychiat 2013;203:373–80. 10.1192/bjp.bp.112.114405 24072756

[14] Kroenke K Spitzer RL Williams JB The PHQ-15: validity of a new measure for evaluating the severity of somatic symptoms. Psychosom Med 2002;64:258–66. 11914441 10.1097/00006842-200203000-00008

[15] Katon WJ Walker EA Medically unexplained symptoms in primary care. J Clin Psychiat 1998;59:15–21. 9881537

[16] Libby AM Ghushchyan V McQueen RB Economic grand rounds: psychological distress and depression associated with job loss and gain: the social costs of job instability. Psychiatr Serv 2010;61:1178–80. 21123399 10.1176/ps.2010.61.12.1178

[17] Han X Lin CC Li C Association between serious psychological distress and health care use and expenditures by cancer history. Cancer 2015;121:614–22. 25345778 10.1002/cncr.29102 PMC4492528

[18] Liu LF Ge HM Cui KY A cross-sectional study on depressive disorder and related factors in medical inpatients. J Psychiat 2008;21:244–7.

[19] Mitchell AJ Rao S Vaze A Can general practitioners identify people with distress and mild depression? meta-analysis of clinical accuracy. J Affect Disord 2011;130:26–36. 20708274 10.1016/j.jad.2010.07.028

[20] Jiang RH Dang WM Ma H Recognition of depression and related risk factors among non psychiatric doctors in tertiary general hospital outpatients in Beijing. Zhonghua Nei Ke Za Zhi 2010;49:477–9. 20979732

[21] Yan ZY Gu MJ Zhong BL Prevalence, risk factors and recognition rates of depressive disorders among inpatients of tertiary general hospitals in Shanghai, China. J Psychosom Res 2013;75:65–71. 23751241 10.1016/j.jpsychores.2013.03.003

[22] Husted JR Insight in severe mental illness: implications for treatment decisions. J Am Acad Psychiatry Law 1999;27:33–49. 10212025

[23] De las Cuevas C Rivero-Santana A Perestelo-Pérez L Attitudes toward concordance in psychiatry: a comparative, cross-sectional study of psychiatric patients and mental health professionals. BMC Psychiatry 2012;30:53. 10.1186/1471-244X-12-53 PMC3403848 22646974

[24] Kerse N Buetow S Mainous AG Physician-patient relationship and medication compliance: a primary care investigation. Ann Fam Med 2004;2:455–61. 15506581 10.1370/afm.139 PMC1466710

[25] Li J Ma H He YL Mental health system in China: history, recent service reform and future challenges. World Psychiatry 2011;10:210–6. 21991281 10.1002/j.2051-5545.2011.tb00059.x PMC3188776

[26] Kroenke K Spitzer RL Williams JBW The PHQ-9: validity of a brief depression severity measure. J Gen Intern Med 2001;16:606–13. 11556941 10.1046/j.1525-1497.2001.016009606.x PMC1495268

[27] Kroenke K Spitzer RL Williams JB The patient health questionnaire somatic, anxiety, and depressive symptom scales: a systematic review. Gen Hosp Psychiat 2010;32:345–59. 10.1016/j.genhosppsych.2010.03.006 20633738

[28] Manea L Gilbody S McMillan D Optimal cut-off score for diagnosing depression with the Patient Health Questionnaire (PHQ-9): a meta-analysis. CMAJ 2012;184:E191–6. 22184363 10.1503/cmaj.110829 PMC3281183

[29] Bian CD He XY Qian J The reliability and validity of a modified patient health questionnaire for screening depressive syndrome in general hospital outpatient. J Tongji Univ (Med Sci) 2009;30:136–40.

[30] Kroenke K Spitzer RL Williams JB Anxiety disorders in primary care: prevalence, impairment, comorbidity, and detection. Ann Intern Med 2007;146:317–25. 17339617 10.7326/0003-4819-146-5-200703060-00004

[31] Witthöft M Hiller W Loch N The Latent structure of medically unexplained symptoms and its relation to functional somatic syndromes. Int J Behav Med 2013;20:172–83. 22618306 10.1007/s12529-012-9237-2

[32] He XY Li CB Qian J Reliability and validity of a generalized anxiety disorder scale in general hospital outpatients. Shanghai Arch Psychiatry 2010;22:200–3.

[33] Korber S Frieser D Steinbrecher N Classification characteristics of the Patient Health Questionnaire-15 for screening somatoform disorders in a primary care setting. J Psychosom Res 2011;71:142–7. 21843748 10.1016/j.jpsychores.2011.01.006

[34] Lee S Ma YL Tsang A Psychometric properties of the Chinese 15-item Patient Health Questionnaire in the general population of Hong Kong. J Psychosom Res 2011;71:69–73. 21767685 10.1016/j.jpsychores.2011.01.016

[35] Fink P Ewald H Jensen J Screening for somatisation and hypochondriasis in primary care and neurological in-patients: a seven-item scale for hypochondriasis and somatisation. J Psychosom Res 1999;46:261–73. 10193917 10.1016/s0022-3999(98)00092-0

[36] Conradt M Cavanagh M Franklin J Dimensionality of the Whiteley Index: assessment of hypochondriasis in an Australian sample of primary care patients. J Psychosom Res 2006;60:137–43. 16439266 10.1016/j.jpsychores.2005.07.003

[37] Hansen MS Fink P Frydenberg M Complexity of care and mental illness in medical inpatients. Gen Hosp Psychiat 2001;23:319–25. 10.1016/s0163-8343(01)00162-1 11738462

[38] Hansen MS Fink P Søndergaard L Mental illness and health care use: a student among new neurological patients. Gen Hosp Psychiat 2005;27:119–24. 10.1016/j.genhosppsych.2004.10.005 15763123

[39] Clarke DM Piterman L Byrne CJ Somatic symptoms, hypochondriasis and psychological distress: a study of somatisation in Australian general practice. Med J Aust 2008;189:560–4. 19012554 10.5694/j.1326-5377.2008.tb02180.x

[40] Lee S Ng KL Ma YL A general population study of the Chinese Whiteley-7 Index in Hong Kong. J Psychosom Res 2011;71:387–91. 22118380 10.1016/j.jpsychores.2011.05.013

[41] Wong FK Lam YK Poon A Depression literacy among Australians of Chinese-speaking background in Melbourne, Australia. BMC Psychiatry 2010;19:7. 10.1186/1471-244X-10-7 PMC2827400 20082724

[42] Chan B Parker G Some recommendations to assess depression in chinese people in Australasia. Aust NZ J Psychiat 2004;38:141–7. 10.1080/j.1440-1614.2004.01321.x 14961932

[43] Ying YW Explanatory models of major depression and implications for help-seeking among immigrant Chinese-American women. Cult Med Psychiatry 1990;14:393–408. 2245642 10.1007/BF00117563

[44] Ridd M Lewis G Peters TJ Detection of patient psychological distress and longitudinal patient–doctor relationships. Br J Gen Pract 2012;3:e167–73. 10.3399/bjgp12X630052 PMC3289822 22429433

